# Metabolomic Associations of Asthma in the Hispanic Community Health Study/Study of Latinos

**DOI:** 10.3390/metabo12040359

**Published:** 2022-04-16

**Authors:** Yura Lee, Han Chen, Wei Chen, Qibin Qi, Majid Afshar, Jianwen Cai, Martha L. Daviglus, Bharat Thyagarajan, Kari E. North, Stephanie J. London, Eric Boerwinkle, Juan C. Celedón, Robert C. Kaplan, Bing Yu

**Affiliations:** 1 Department of Epidemiology, Human Genetics and Environmental Sciences, School of Public Health, The University of Texas Health Science Center at Houston, Houston, TX 77030, USA; yura.lee@uth.tmc.edu (Y.L.); han.chen.2@uth.tmc.edu (H.C.); eric.boerwinkle@uth.tmc.edu (E.B.); 2 Department of Pediatrics, School of Medicine, University of Pittsburgh, Pittsburgh, PA 15224, USA; wec47@pitt.edu (W.C.); juan.celedon@chp.edu (J.C.C.); 3 Department of Epidemiology & Population Health, Albert Einstein College of Medicine, Bronx, NY 10461, USA; qibin.qi@einsteinmed.org; 4 Department of Medicine, University of Wisconsin School of Medicine and Public Health, Madison, WI 53726, USA; majid.afshar@wisc.edu (M.A.); robert.kaplan@einsteinmed.org (R.C.K.); 5 Department of Biostatistics, University of North Carolina at Chapel Hill, Chapel Hill, NC 27516, USA; cai@bios.unc.edu; 6 Institute of Minority Health Research, University of Illinois College of Medicine, Chicago, IL 60612, USA; daviglus@uic.edu; 7 Department of Laboratory Medicine and Pathology, University of Minnesota, MMC 609, 420 Delaware Street, Minneapolis, MN 55455, USA; thya0003@umn.edu; 8 Department of Epidemiology and Carolina Center for Genome Sciences, School of Public Health, University of North Carolina at Chapel Hill, Chapel Hill, NC 27514, USA; kari_north@unc.edu; 9 Department of Health and Human Services, National Institute of Environmental Health Sciences, National Institutes of Health, Research Triangle Park, NC 27709, USA; london2@niehs.nih.gov; 10 Division of Pulmonary Medicine, Children’s Hospital of Pittsburgh of UPMC, Pittsburgh, PA 15224, USA

**Keywords:** asthma, metabolites, metabolomics, HCHS/SOL, Hispanics, 1-arachidonoyl-GPA (20:4)

## Abstract

Asthma disproportionally affects Hispanic and/or Latino backgrounds; however, the relation between circulating metabolites and asthma remains unclear. We conducted a cross-sectional study associating 640 individual serum metabolites, as well as twelve metabolite modules, with asthma in 3347 Hispanic/Latino background participants (514 asthmatics, 15.36%) from the Hispanic/Latino Community Health Study/Study of Latinos. Using survey logistic regression, per standard deviation (SD) increase in 1-arachidonoyl-GPA (20:4) was significantly associated with 32% high odds of asthma after accounting for clinical risk factors (*p* = 6.27 × 10^−5^), and per SD of the green module, constructed using weighted gene co-expression network, was suggestively associated with 25% high odds of asthma (*p* = 0.006). In the stratified analyses by sex and Hispanic and/or Latino backgrounds, the effect of 1-arachidonoyl-GPA (20:4) and the green module was predominantly observed in women (OR = 1.24 and 1.37, *p* < 0.001) and people of Cuban and Puerto-Rican backgrounds (OR = 1.25 and 1.27, *p* < 0.01). Mutations in Fatty Acid Desaturase 2 (*FADS2*) affected the levels of 1-arachidonoyl-GPA (20:4), and Mendelian Randomization analyses revealed that high genetically regulated 1-arachidonoyl-GPA (20:4) levels were associated with increased odds of asthma (*p* < 0.001). The findings reinforce a molecular basis for asthma etiology, and the potential causal effect of 1-arachidonoyl-GPA (20:4) on asthma provides an opportunity for future intervention.

## 1. Introduction

Asthma is a common respiratory disease that affects more than 25 million people in the United States [1]. Although Hispanic/Latino individuals have a lower prevalence of asthma than white or black individuals [2,3], there is wide variability in asthma morbidity among Hispanic and/or Latino backgrounds. In the USA, the burden of asthma is highest in Puerto Ricans and lowest in Mexican Americans [4,5]. Asthma is a complex multifactorial disease [4,6]. Differences in socioeconomic status, tobacco use, air pollution, and obesity are associated with ethnic disparities in asthma [5,7], but the mechanisms underlying these associations are not fully understood [8,9].

Metabolomics can be used to uncover causal pathways and biomarkers of asthma and asthma-related phenotypes [10,11,12]. Indeed, multiple circulating metabolites (mainly involved in the pathways of inflammation, immunity, lipid, oxidative stress, hypoxia response metabolism, and tricarboxylic acid cycle) have been linked to asthma in people of non-Hispanic/Latino backgrounds [10,12,13,14,15,16,17,18]. Some of them found that the metabolites from arachidonic acid metabolism were associated with asthma [15,16,17,18]. However, there have been few studies conducted on Hispanic and/or Latino backgrounds. In one study, it was discovered that metabolites in glycerophospholipid, linoleic acid, and pyrimidine metabolism were associated with percent-predicted forced expiratory volume in one second/forced vital capacity ratio (FEV1/FVC), FEV1/FVC post-bronchodilator, and airway hyper-responsiveness (AHR) in Costa Rican children with asthma [19].

To date, there has been no large-scale study of metabolites and asthma in adult Hispanic and/or Latino backgrounds, the fastest growing population in the USA. To this end, we examined the relationship between biologically informative metabolites and asthma in 3347 subjects who participated in the Hispanic/Latino Community Health Study/Study of Latinos (HCHS/SOL). The findings of the present study suggest candidate pathways for asthma in Hispanic/Latino populations in general, and in two Hispanic/Latino backgrounds in particular.

## 2. Results

### 2.1. Study Sample Characteristics

A total of 3347 participants consisting of 514 asthmatics and 2833 non-asthmatics were selected for the study. Characteristics of the study sample are described in Table 1. There was no significant difference in age between asthmatics (47.14 ± 13.40 years) and non-asthmatics (45.93 ± 13.37 years) (*p* = 0.060). Participants with asthma were more likely to be female, born in the USA with a longer-living period in the USA, had a smoking history, lower family income, and higher BMI compared to those without asthma. Those who were from Puerto-Rican and Cuban backgrounds occupied considerable portions within asthmatics in comparison with the other Hispanic and/or Latino backgrounds (Puerto-Rican, 40.47%; Cuban, 21.21%). There were no differences in education and lipid levels between asthmatics and non-asthmatics. When comparing pulmonary function and additional risk factors between asthmatics and non-asthmatics, it showed that asthmatics had poorer pulmonary function compared to non-asthmatics (Table S1).

### 2.2. Single Metabolites and Asthma

**Figure 1:**
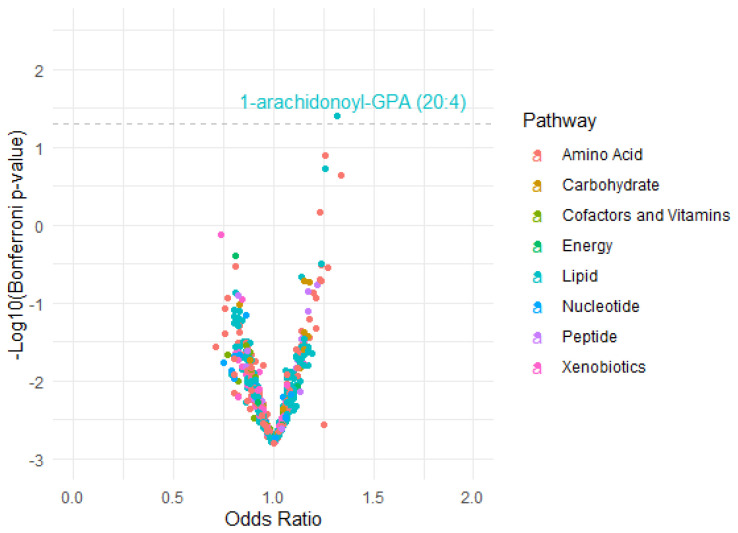
Volcano plot illustrating the relationship between 640 single metabolites and asthma. The odds ratios were calculated from survey logistic regressions adjusting for age, sex, immigration status, field centers, years of living in the U.S., Hispanic and/or Latino backgrounds, low-density lipoprotein cholesterol (LDL), high-density lipoprotein cholesterol (HDL), triglycerides (TG), smoking, education level, and household income. *x*-axis: odds ratio between each metabolite level and asthma status; *y*-axis: −log_10_(Bonferroni adjusted *p*-value) for each metabolite with the dashed line (−log_10_0.05 = 1.301) representing the significance threshold.

Three metabolites, 1-arachidonoyl-GPA (20:4), glutamate, and tyrosine, were significantly associated with asthma in Model 1 with basic demographic factors adjusted (Table 2). With further adjustment of other risk factors, the effects for most metabolites were slightly attenuated. Only 1-arachidonoyl-GPA (20:4), a lysophospholipid, manifested statistical significance across all three models with similar effect sizes. The estimated odds for asthma were 1.32 (95% CI: 1.15–1.51) per one SD increase in 1-arachidonoyl-GPA (20:4) with full covariates adjustment, and the effect remained unchanged in the sensitivity analysis using doctor-diagnosed asthma (data not shown). Figure 1 visualized the distribution of the effect of Model 3 for 640 single metabolites.

### 2.3. Metabolite Modules and Asthma

In addition to single metabolite asthma associations, metabolite modules were analyzed to explore the potential metabolic pathways in relation to asthma. Twelve different metabolite modules with unique colors were generated, and the number of metabolites included in each module was ranged from 13 (green-yellow) to 191 (grey) (Figure S1, Table S2).

**Figure 2:**
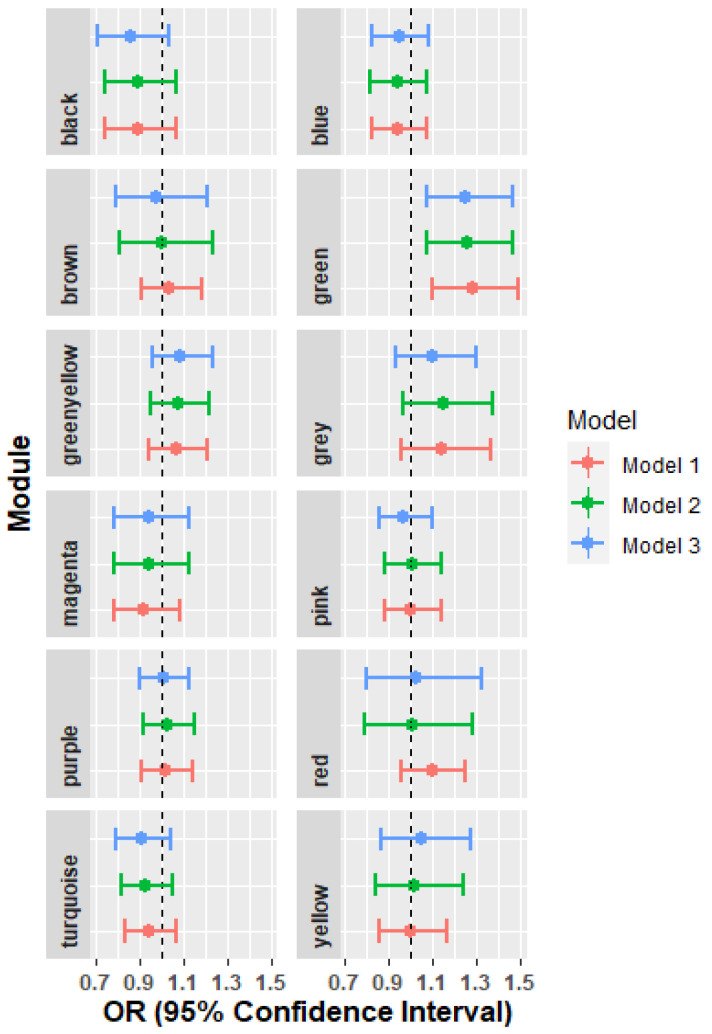
Forest plot representing odds ratio and 95% confidence interval of three models between each module and asthma. Model 1 included age, sex, immigration status, field centers, years of living in the U.S., and Hispanic and/or Latino backgrounds; Model 2 additionally adjusted for LDL, HDL, and TG; and Model 3 supplemented smoking, education level, and household income.

After applying three consecutive models, none of the modules showed significant associations in the fully adjusted model (Table S3). However, the green module was significantly associated with asthma in the demographics adjusted model, and its effect became slightly attenuated in the fully adjusted model (Model1: OR = 1.28, 95% CI: 1.10–1.49; Model 2: OR = 1.25, 95% CI: 1.07–1.47; Model 3: OR = 1.25, 95% CI: 1.07–1.46) (Figure 2). This trend was not altered in the sensitivity analyses (data not shown). When correlating with clinical risk factors, the green module appeared to have modest correlation with BMI (r = 0.22), TG (r = 0.29), pre- and post- bronchodilator values of FEV1 and FVC (r = 0.24 to 0.33), whereas the red and yellow modules had relatively higher relationship with TG and LDL respectively (Red: TG (r = 0.86); Yellow: LDL (r = 0.68)) (Figure S2).

The green module included 40 metabolites comprised of four super-pathways: amino acid (26), peptide (12), lipid (1), and nucleotide (1) (Table S4). About half of the metabolites (47.5%) showed suggestive association with asthma in the fully adjusted model (*p* < 0.05). Among those 40 metabolites, there was no significant pathway overrepresented based on Bonferroni adjusted *p*-value, while two pathways showed nominal significance (Valine, Leucine, and Isoleucine Degradation: *p* = 0.004; Phenylalanine and Tyrosine Metabolism: *p* = 0.016) (Figure S3, Table S5).

### 2.4. Stratification Analysis by Sex and Hispanic/Latino High-Risk Backgrounds

In the stratified analysis to explore potential effect modification by sex and Hispanic/Latino high-risk backgrounds, about 36–41% increases in odds were observed per one SD increase in the green module eigenvector in women (*p* < 0.001) (Table 3 and Table S6). In contrast, no significant increase in odds was observed in men. Meanwhile, about a 26–27% increase in odds was observed in the high-risk group of Cubans and Puerto-Ricans (*p* < 0.01), but the effect was weakened in the other Hispanic and/or Latino backgrounds when adjusting all risk factors shown in Model 3. As for 1-arachidonoyl-GPA (20:4), similar patterns were found that the effect sizes were larger in women and Cuban and Puerto-Rican backgrounds compared to men and the other Hispanic/Latino backgrounds. The effects were consistent across three models in women and men (OR = 1.22–1.24, *p* < 0.001). Moreover, the larger odds of asthma were seen in people of Cuban and Puerto-Rican backgrounds (OR = 1.25, 95% CI = 1.09–1.49, *p* < 0.01) in comparison with the other Hispanic/Latino backgrounds (OR = 1.15, 95% CI = 1.00–1.32, *p* < 0.05). A two-sided z score test revealed statistical significance for the interaction effects of sex and Hispanic/Latino high-risk backgrounds on asthma (Table S7).

### 2.5. Causal Effect Exploration

For the one asthma-related metabolite, 1-arachidonoyl-GPA (20:4), one locus of rs28456, an intronic variant of Fatty Acid Desaturase 2 (*FADS2*), was previously reported in HCHS/SOL that reached genome-wide significance for asthma (beta = 0.16, se = 0.03, p = 6.08 × 10^−11^, allele frequency of the effect allele A = 0.54) [20]. Rs28456 showed a strong direction association with adult asthma, but a modest association with childhood asthma in European populations; and it was also associated with adult asthma in East Asian populations [21,22]. In all scenarios, Mendelian Randomization analyses suggested that high genetically regulated 1-arachidonoyl-GPA (20:4) levels were associated with an increased risk of asthma (Table 4).

## 3. Discussion

In a cross-sectional analysis of 640 single metabolites in 3347 participants in HCHS/SOL, 1-arachidonoyl-GPA (20:4) was significantly associated with asthma status. The green module, consisting of 40 metabolites, also showed a positive association with asthma though the effect was attenuated with further adjustment of clinical risk factors. The estimated effects of 1-arachidonoyl-GPA (20:4) and the green module were found predominantly in women and participants with Cuban and Puerto Rican backgrounds. Mendelian Randomization analyses revealed a potential causal association between 1-arachidonoyl-GPA (20:4) and asthma.

1-arachidonoyl-GPA (20:4), a metabolite in the lysophospholipid pathway, is a derivative of arachidonic acid that plays a key role in inflammation [20]. Arachidonic acid is a precursor for a diverse range of lipid inflammatory mediators that may cause airway inflammation in asthma by generating proinflammatory mediators [23,24]. Several arachidonic metabolites have been reported for their associations with asthma [15,16,17,18]. Leukotriene B4 (LTB4) and 5-hydroxyeicosatetraenoic acid (5-HETE) are mediators generated by alveolar macrophages in lung inflammation [25]. The activities of the metabolites related to cysteinyl leukotrienes (CysLT), such as leukotriene C4 (LTC4) and secretory phospholipases A2 (sPLA2), are enhanced in asthma [26]. A more recent study found that prostaglandin E2 (PGE2), 15-Deoxy-Delta-12,14-PGJ2 (15d-PGJ2), and lipoxins (LXs) are good candidates to develop asthma treatments [27], but those studies did not focus on Hispanic and/or Latino backgrounds. One recent study analyzed metabolites with lung function parameters in asthmatic children in Costa Rica, and found out that the metabolites of glycerophospholipid, linoleic acid, and pyrimidine metabolism were related to asthma severity [19]. In the present study, some glycerophospholipids and metabolites in pyrimidine metabolism, e.g., 1-lignoceroyl-GPC (24:0), and 5-methyluridine (ribothymidine), were associated with asthma at a nominal significance level (*p* < 0.05), but did not meet the significance threshold after applying stringent Bonferroni correction to account for multiple testing. Further investigation is warranted to detail the associations between those metabolites and asthma.

The asthma-related green module consisted of 40 metabolites: 65% were under the amino acid pathway and 30% were under the peptide pathway. Interestingly, 1-arachidonoyl-GPA (20:4) was not included in the green module, highlighting the importance of considering metabolite pathways, in addition to single metabolites, when studying the metabolic effects of a disease such as asthma. Of the 26 metabolites categorized into amino acids, twelve of which were branched-chain amino acids (BCAAs), classified as leucine, isoleucine, and valine metabolism. BCAAs as the essential amino acids have a number of biological functions for energy, stress, and muscle metabolism. BCAAs have been identified as biomarkers for insulin resistance and type 2 diabetes [28], but there are few studies about BCAAs’ role in asthma pathophysiology. Matysiak et al. [29] described the decreased level of valine, one of the BCAAs, in asthmatic children (*n* = 13) compared to healthy ones (*n* = 17). Another study showed considerably lower BCAA levels in asthmatics with a low fraction of exhaled NO (F_E_NO) (*n* = 9), a biomarker of eosinophilic airway inflammation, than in those with high F_E_NO or in healthy controls (*n* =19) [30]. Our findings underpin those prior findings in that BCAAs may be a key metabolic pathway in asthma.

Twelve metabolites from the green module belonged to Gamma-glutamyl Amino Acids (GGAAs). They are catalyzed by gamma-glutamyl transferase (GGT). A few studies have found associations between asthma and GGT or GGT-related metabolites. The level of GGT in serum is inversely linked to pulmonary function [31]; however, the study was limited to chronic obstructive pulmonary disease (COPD). Inhibiting GGT activity in lung lining fluid has been developed as a novel target to treat asthma [32,33].

Additionally, the red and yellow modules were examined closely as they showed relatively higher relationships with TG and LDL respectively compared to the other modules in Figure S2. The red module is composed of 21 metabolites. Nineteen of them (90%) are under the lipid pathway (Sub pathways: ten in monoacylglycerol and nine in diacylglycerol), and two of them (10%) are under the cofactors and vitamins pathway (Sub pathway: two in tocopherol metabolism). One of the major pathways for TG synthesis is the acylation of monoacylglycerol by monoacylglycerol acyltransferase enzymes to form diacylglycerol [34,35], therefore suggesting a high association with TG. The yellow module consists of 44 metabolites. All of them are under the lipid pathway (Sub pathways: 33 in sphingolipid metabolism, 10 in ceramides, and 1 in sterol). Sphingolipids are a group of lipids, containing a molecule of especially ceramides and sphingomyelins [36], and are known to play crucial roles in maintaining membrane function and integrity, preserving lipoprotein structure and functions [37]. Sphingomyelin is the most prevalent sphingolipid found in lipoproteins, and VLDL/LDL and HDL account for around 63–75% and 25–35% of sphingomyelin, respectively [38]. We also observed that the yellow module, consisting of various sphingomyelins and ceramides, showed a strong relationship with LDL.

The estimated effects of 1-arachidonoyl-GPA (20:4) and the green module were modified by sex or Hispanic/Latino backgrounds. Although ethnicity is a potential effect modifier in asthma [39], this has not been shown for Hispanic and/or Latino backgrounds in a large study of metabolites or metabolite clusters and asthma. Effect modification by Hispanic and/or Latino backgrounds is likely to be predominantly due to underlying differences in risk factors correlated with social determinants of health (e.g., air pollution or diet) [40,41,42].

Two-sample Mendelian Randomization (MR) has been used to explore underlying causal associations between an exposure and an outcome. It is suboptimal to perform MR if exposure and outcome data were extracted from different ethnic populations, therefore, observing the direct association between asthma and the locus of 1-arachidonoyl-GPA (20:4) is an alternative approach to exploring casualty [43]. In this study, genetically highly regulated 1-arachidonoyl-GPA (20:4) levels were observed to be associated with asthma. This is consistent with an observational study that showed that high levels of 1-arachidonoyl-GPA (20:4) were correlated with asthma, and 1-arachidonoyl-GPA (20:4) is influenced by *FADS2* [20]. *FADS2* has been linked to adult asthma in European backgrounds [21,22], and key inflammatory metabolites have been identified near *FADS2* [44]. Moreover, decreased activity of *FADS2* is accompanied by asthma progression [45], which might be caused by the interrupted metabolism of polyunsaturated fatty acids (PUFAs) and pro-resolving lipid mediator synthesis.

We recognized several study limitations. First, we only explored known metabolites with a low missing rate. The full picture of the metabolic effect on asthma warrants future investigation. Second, other lifestyle factors, such as diet, may influence asthma and metabolites [46,47]. How metabolites may mediate the effect of diet on asthma remain to be explored. Moreover, genetic factors—such as family history of asthma—and air pollution as an environmental risk factor [48,49] were not analyzed in the present study; thus, it is recommended for future research to demonstrate a more robust association between the identified metabolite and the metabolite module, and asthma in Hispanic and/or Latino backgrounds. Third, it would be worth future investigation of the impact of asthma treatment, such as inhaled corticoids, on circulating metabolites, because its use might change the biological metabolomic profiles associated with asthma [50]. Fourth, self-reported asthma cases were used in the present study, which might not reflect the true disease status. A sensitivity analysis comparing doctor diagnosed to self-reported asthma cases did not alter our main findings. Fifth, few large Hispanic/Latino background studies with metabolomic profiling data are available, therefore, we were not able to perform external validation. However, the MR analysis using published genetic summary statistics from external studies demonstrated the potential causal effect of 1-arachidonoyl-GPA (20:4) on asthma, strengthening the observed association. Lastly, the current study used a cross-sectional design, which limited the ability to estimate temporal relationships between metabolites and asthma. However, we identified a potential causal association between 1-arachidonoyl-GPA (20:4) and asthma by leveraging genetic summary statistics from genome-wide association studies.

## 4. Materials and Methods

### 4.1. Study Samples

Subject recruitment and the study design of HCHS/SOL have been previously described in detail [51,52]. In brief, the HCHS/SOL is a prospective cohort study aiming to identify factors influencing the health of Hispanic and/or Latino backgrounds. By using a stratified two-stage area probability sampling method in four communities in the US (Chicago, IL; Miami, FL; Bronx, NY, and San Diego, CA), participants aged 18 to 74 years at the screening were recruited from randomly selected households. In total, 16,415 individuals who self-identified as Hispanic and/or Latino backgrounds (South Americans, Central Americans, Mexicans, Puerto Ricans, Cubans, and Dominicans) were recruited between June 2008 and July 2011. Of those completing the first study visit, 3349 randomly chosen participants had metabolite measures and complete clinical data for this study. After removing two outlier samples, 3347 participants were included in the current analysis. The HCHS/SOL was approved by the institutional review boards at each participating institution, and written informed consent was obtained from all study participants.

### 4.2. Metabolite Profiling

Fasting serum samples were collected from the HCHS/SOL baseline visit for metabolomic profiling and stored at −70 °C since collection. The profiling was performed at Metabolon (Durham, NC, USA) using the Discovery HD4 platform in 2017 [20]. Untargeted liquid chromatography–mass spectrometry (LC-MS) protocol was utilized to semi-quantify metabolites [53,54,55]. In total, 1136 metabolites were discovered, including 782 known and 354 unknown metabolites. Finally, 640 analyzable metabolites were verified as only known metabolites with missing rates ≤ 25% were regarded for quality control. Missing data for the metabolites were imputed to half of the lowest value [56,57]. Additional details are provided in the Supplemental Methods (Table S8).

### 4.3. Ascertainment of Asthma and Covariates

In HCHS/SOL, asthma cases were identified using questionnaire data. A case for the current study was defined as those who answered “yes” to the survey question, “Have you ever had asthma?” [7,58]. A cross-check was conducted to verify that all of the cases diagnosed by medical professionals in the present study were counted as cases in the case definition question. All self-defined cases as ever-asthma cases included those diagnosed by health professionals. The non-asthmatics were defined as those who neither reported ever asthma nor were diagnosed with asthma by a physician. A total of 514 individuals were characterized as asthma cases and 2833 were controls. There were 23 cases (4.47%) of self-reported ever asthma cases that were not diagnosed by medical professionals. A sensitivity analysis was conducted to evaluate the consistency between the self-reported and doctor-diagnosed asthma definitions.

Risk factors for asthma were collected from the baseline survey questionnaires including age, sex, smoking status (never; former; current), cigarette years, education levels (less than high school; high school or equivalent; greater than high school or equivalent), annual household income (ten categories in total; from less than $10,000 to $29,999 by $5000, from $30,000 to $50,000 by $10,000, and from 50,001 to more than $100,000 by $25,000), immigrant status (residence period in US and US born or immigrant), and self-defined Hispanic and/or Latino backgrounds (Dominican; Central American; Cuban; Mexican; Puerto-Rican; South American) [59,60,61,62,63]. TG and HDL levels were measured with the serum from 12-hour fasting blood samples collected in accordance with standard protocols [64], and the Friedewald equation was employed to calculate LDL levels [65]. Body Mass Index (BMI) was calculated as weight in kilograms divided by height in meters squared [66]. Pulmonary function measures, including FEV1, FVC, and FEV1/FVC ratio of pre and post bronchodilator levels were gauged using a dry rolling seal spirometer with automated quality checks by American Thoracic Society and European Respiratory Society guidelines [67,68]. Eosinophil counts were enumerated by Sysmex XE-2100 instrument (Sysmex America) at the University of Minnesota based on national and international standards and procedures with the whole blood in EDTA collected at the baseline examination [69].

### 4.4. Statistical Analysis

The demographic characteristics of the samples between asthmatics and non-asthmatics were compared using a t-test for continuous variables and a chi-square test for categorical variables. Each of 640 single metabolites was tested for the association with asthma using a survey logistic regression analysis, incorporating sampling weights in the statistical models [70]. Three consecutive models were performed: Model 1 included age, sex, immigration status, field centers, and Hispanic and/or Latino backgrounds; Model 2 additionally adjusted for LDL, HDL, and TG; and Model 3 supplemented smoking, education level, and household income.

We constructed metabolite modules based on similarities using Weighted Gene Co-expression Network Analysis (WGCNA) [71]. It is used to locate clusters, called modules, of highly correlated genes, metabolites, or proteins [72]. The soft-thresholding power β was computed and selected as 5, which was the first number of the degree of independence exceeding 0.9 with soft thresholding r^2^ of 0.928 (Figure S4, Table S9). The algorithm identified the co-expressed metabolite modules with a minimum module size of 10. A dissimilarity matrix was used to distinguish modules through a dynamic tree-cutting algorithm by splitting the whole network into multiple co-expressed modules. Random colors were assigned to the identified modules. The modules were considered to be merged with similar modules based on the height cut criteria of 0.25, implying the correlation between modules was 0.75 [73,74,75]. Metabolites not showing similarity with any clusters were classified into the grey module. Module eigenvectors were calculated as the first principal component of the expression matrix of the corresponding module, and were standardized before analyses [76,77,78]. The eigenvectors were analyzed using the same aforementioned three models. Bonferroni adjusted *p*-values < 0.05 were considered statistically significant for both single metabolites and metabolite modules analyses.

The Pearson correlations between each module’s eigenvectors and risk factors: age, cigarette years, eosinophils, pulmonary function (FEV1, FVC, and FEV1/FVC ratio of pre and post respectively), BMI, and lipids (LDL, HDL, and TG) were estimated after demonstrating the different distribution between asthmatics and non-asthmatics by t-test. [79]. Since biological sex plays a key role in asthma [80], and individuals with Puerto-Rican and Cuban backgrounds show more prevalent asthma compared to people of other Hispanic/Latino backgrounds [58,81,82], stratified analyses were conducted to determine the potential effect modification of sex and Hispanic/Latino high-risk backgrounds on metabolite and metabolite module associations with asthma. Additionally, a two-sided z score test was computed to test interaction effects on asthma between sex and Hispanic/Latino high-risk backgrounds, and metabolite and metabolite modules, respectively.

For the identified asthma-related metabolite module, over-representation analysis (ORA) was performed in MetaboAnalyst 5.0 to identify biologically meaningful metabolome patterns [83]. ORA is designed to test what biological pathways would be represented more often than expected by chance [84]. A total of 40 metabolites chosen based on WGCNA and grouped in a module were fed into the pathway database of the Human Metabolome Database (HMDB); 37 metabolites were successfully mapped and were carried over into ORA. The Bonferroni adjusted *p*-value < 0.05 was defined as significant accounting for 34 pathways tested.

For the metabolite associated with asthma, its significant genetic loci were looked up in the published metabolite genome-wide association study from HCHS/SOL (*p* < 1.23 × 10^−10^) [20]. The direct association between the metabolite loci and asthma was examined primarily using published asthma genome-wide association studies from European and East Asian populations [21,22]. The MR approach was applied secondarily to assess their potential causal relation, since using ethnic different populations for exposure and outcome were suboptimal for MR. The MR analysis was performed using the R package “TwoSampleMR” (version 0.5.6).

All analyses were conducted using R 4.0.5, and statistical significance was defined as a two-sided *p*-value < 0.05 unless specified otherwise.

## 5. Conclusions

In summary, we identified 1-arachidonoyl-GPA (20:4) and a metabolite module that were associated with asthma respectively in Hispanic and/or Latino backgrounds. Our findings provide additional insights into asthma etiology and candidates for future more targeted metabolomic studies on asthma.

## Figures and Tables

**Table 1:** Demographic Characteristics of the Samples in Study (*n* = 3347).

	Asthma (*n* = 514)	Non-Asthma (*n* = 2833)	*p*-Value
Female, N (%)	343 (66.73)	1561 (55.10)	<0.001
Age, years ± SD	47.14 ± 13.40	45.93 ± 13.37	0.060
Ethnicity, N (%)			<0.001
Dominican	42 (8.17)	286 (10.10)	
Central American	47 (9.14)	286 (10.10)	
Cuban	109 (21.21)	445 (15.71)	
Mexican	93 (18.08)	1216 (42.92)	
Puerto-Rican	208 (40.47)	404 (14.26)	
South American	15 (2.92)	196 (6.92)	
Cigarette Use, N (%)			<0.001
Never	247 (48.05)	1710 (60.36)	
Former	117 (22.76)	566 (19.98)	
Current	150 (29.18)	557 (19.66)	
Less than High School Education, N (%)	174 (33.98)	970 (34.23)	0.905
BMI, kg/m^2^ ± SD	31.48 ± 7.12	29.58 ± 5.90	<0.001
Lipids, mg/dL ± SD			
LDL	121.53 ± 37.27	123.96 ± 36.68	0.173
HDL	49.56 ± 13.34	49.70 ± 13.04	0.826
TG	128.20 ± 65.38	129.93 ± 68.39	0.585
Immigration Status			
Residence Period in US, years ± SD	28.06 ± 16.57	22.24 ± 14.98	<0.001
US Born, N (%)	134 (26.07)	463 (16.34)	<0.001
Annual Family Income, N (%)			
<$20,000	294 (57.20)	1306 (46.10)	<0.001

Definition of abbreviations: SD = Standard Deviation; LDL = Low-Density Lipoprotein; HDL = High-Density Lipoprotein; TG = Triglyceride; BMI = Body Mass Index. Results are presented as mean ± SD or number (%) of persons as appropriate.

**Table 2:** Logistic Regression Analysis of 640 Single Metabolites.

Metabolite	Model (1)	Model (2)	Model (3)
1-arachidonoyl-GPA (20:4)	1.32 * (1.16, 1.51)	1.33 * (1.17, 1.52)	1.32 * (1.15, 1.51)
Glutamate	1.36 * (1.17, 1.58)	1.36 (1.16, 1.59)	1.34 (1.14, 1.57)
Tyrosine	1.28 * (1.13, 1.44)	1.27 (1.12, 1.43)	1.26 (1.12, 1.42)

Odds ratio with 95% confidence interval in parentheses. Bonferroni adjusted *p*-values: * *p* < 0.05. Model 1 included age, sex, immigration status, field centers, years of living in the U.S., and Hispanic and/or Latino backgrounds; Model 2 additionally adjusted for LDL, HDL, and TG; and Model 3 supplemented smoking, education level, and household income.

**Table 3:** Stratification Analysis of Green Module and 1-arachidonoyl-GPA (20:4) by Sex and Hispanic/Latino Backgrounds.

		Cases/Controls	Model (1)	Model (2)	Model (3)
Sex					
Green Module	Women	343/1561	1.41 *** (1.22, 1.64)	1.36 *** (1.17, 1.59)	1.37 *** (1.17, 1.60)
	Men	171/1272	1.00 (0.83, 1.21)	1.05 (0.87, 1.27)	1.04 (0.86, 1.25)
1-arachidonoyl-GPA (20:4)	Women	343/1561	1.23 *** (1.09, 1.38)	1.22 *** (1.08, 1.37)	1.24 *** (1.10, 1.40)
	Men	171/1272	1.12 (0.95, 1.32)	1.15 (0.96, 1.35)	1.13 (0.95, 1.34)
Hispanic/Latino Backgrounds					
Green Module	Cuban and Puerto-Rican Backgrounds	317/849	1.27 ** (1.09, 1.47)	1.26 ** (1.08, 1.48)	1.27 ** (1.09, 1.49)
	Others	197/1984	1.21 * (1.02, 1.45)	1.20 * (1.00, 1.44)	1.20 (1.00, 1.44)
1-arachidonoyl-GPA (20:4)	Cuban and Puerto-Rican Backgrounds	317/849	1.26 *** (1.10, 1.44)	1.26 *** (1.10, 1.44)	1.25 ** (1.09, 1.43)
	Others	197/1984	1.14 (0.99, 1.30)	1.15 * (1.00, 1.31)	1.15 * (1.00, 1.32)

Odds ratio with 95% confidence interval in parentheses. *** *p* < 0.001, ** *p* < 0.01, * *p* < 0.05. Model 1 included age, sex, immigration status, field centers, years of living in the U.S., and Hispanic/Latino backgrounds; Model 2 additionally adjusted for LDL, HDL, and TG; and Model 3 supplemented smoking, education level, and household income.

**Table 4:** The association between rs28456 and asthma from observational studies and Mendelian Randomization analysis.

					Direct Association			Mendelian Randomization		
Study	Outcome	Population	N	AF	beta	se	*p*	beta	se	*p*
Ferreira (2019)	Adult asthma	European	327,253	0.69	1.06	0.01	2.5 × 10^−12^	6.53	0.05	<0.001
Ferreira (2019)	Childhood asthma	European	327,253	0.68	1.03	0.01	0.03	6.30	0.08	<0.001
Ishigaki (2020)	Adult asthma	East Asian	209,808	0.61	0.04	0.02	0.02	0.24	0.01	0.02

AF: allele frequency of the A allele; Beta: effect size of the A allele; se: standard error.

## Data Availability

The phenotypic data presented in this study are openly available in dbGaP (accession number: phs000880.v1.p1). The metabolomics data presented in this study available on request through the HCHS/SOL website (https://sites.cscc.unc.edu/hchs).

## Supplementary Materials

The following are available online at https://www.mdpi.com/article/10.3390/metabo12040359/s1, Figure S1: (a) Clustering dendrograms of metabolites; (b) eigengene dendrogram, Figure S2: Module-trait relationship heatmap, Figure S3: Over-representation analysis of the 40 metabolites in green module, Figure S4: (a) Outliers detection by SampleTree function of WGCNA; (b,c) analysis of network topology for a set of soft-thresholding powers, Table S1: Demographic Characteristics of the Samples in Study Used for the Heatmap of the Pearson Correlation Test, Table S2: The number of metabolites in 12 modules, Table S3: Association between colored modules and asthma, Table S4: Pathways and metabolites classification of green module, Table S5: The List of 1-arachidonoyl-GPA (20:4) and 40 Metabolites in Green Module by LC/MS Analysis, Table S6: Over-representation analysis of the 40 metabolites in green module, Table S7: Stratification analysis of green module and 1-arachidonoyl-GPA (20:4) by sex and Hispanic/Latino backgrounds, Table S8: Interaction Effects of green module and 1-arachidonoyl-GPA (20:4) by sex and Hispanic/Latino backgrounds, Table S9: Scale-free metrics resulting from pickSoftThreshold function of WGCNA [20,53,56,57,85,86].
