# Supplementary material for: Metabolomic Associations of Asthma in the Hispanic Community Health Study/Study of Latinos
Source: Metabolites. 2022 Apr 16;12(4):359. doi: 10.3390/metabo12040359 (PMC9028429; doi:10.3390/metabo12040359)
Supplement: Supplementary file 1 [file metabolites-12-00359-s001.zip › metabolites-1649796-supplementary.pdf]

Supplementary Materials

Supplementary methods

Figure S1: (a) Clustering dendrograms of metabolites; (b) eigengene dendrogram

Figure S2: Module-trait relationship heatmap

Figure S3: Over-representation analysis of the 40 metabolites in Green module

Figure S4: (a) Outliers detection by *SampleTree* function of WGCNA;
(b)&(c) analysis of network topology for a set of soft-thresholding powers

Table S1: Demographic Characteristics of the Samples in Study Used for the Heatmap of the Pearson Correlation Test

Table S2: The number of metabolites in 12 modules

Table S3: Association between colored modules and asthma

Table S4: Pathways and metabolites classification of green module

Table S5: Over-representation analysis of the 40 metabolites in green module

Table S6: Stratification analysis of green module and 1-arachidonoyl-GPA (20:4) by sex and Hispanic/Latino backgrounds

Table S7: Interaction Effects of green module and 1-arachidonoyl-GPA (20:4) by sex and Hispanic/Latino backgrounds

Table S8: The List of 1-arachidonoyl-GPA (20:4) and 40 Metabolites in Green Module by LC/MS Analysis

Table S9: Scale-free metrics resulting from *pickSoftThreshold* function of WGCNA

Supplementary methods

Fasting serum samples were collected from the HCHS/SOL baseline visit for metabolomic profiling and stored at -70 °C since collection. The profiling was performed at Metabolon (Durham, NC) using Discovery HD4 platform in 2017 [1].

A. Mass spectrometry analysis

The collected serum samples were extracted with methanol and analyzed using ultra-performance liquid chromatography (UPLC)-MS/MS for non-targeted mass spectrometry (MS) [2]. All techniques used a Waters ACQUITY UPLC and a Thermo Scientific Q-Exactive high resolution/accurate mass spectrometer using heated electrospray ionization (HESI-II) source and an Orbitrap mass analyzer with a 35,000-mass resolution.

Four methods proceeded after the process of drying the sample extracts and rehydrating them in solvents to make the samples compatible [3].

(1) Acidic positive ion conditions: Water and methanol were used to gradient elute the extract from a C18 column (Waters UPLC BEH C18-2.1x100 mm, 1.7 μm) containing 0.05% perfluoropentanoic acid (PFPA) and 0.1% formic acid (FA).

(2) Acidic positive ion conditions: The extract was gradient eluted from the same C18 column using methanol, acetonitrile, water, 0.05% PFPA, and 0.01% FA at a higher total organic content.

(3) Negative ion conditions: For this aliquot, a separate dedicated C18 column was used. Water and methanol were used to gradient elute the extract from the column, and the solution was with 6.5mM Ammonium Bicarbonate at pH 8.

(4) Negative ion conditions: It was analyzed with elution from a HILIC column (Waters UPLC BEH Amide 2.1x150 mm, 1.7 μm) while using a gradient consisting of water and acetonitrile with 10mM Ammonium Formate, pH 10.8. Using dynamic exclusion, the MS analysis alternated between MS and data-dependent MSn (sequential mass spectrometry) scans. The scan range was 70-1000 m/z and varied somewhat across technologies.

B. Identification and Classification of Metabolites

At Metabolon, metabolites were recognized using an automated comparison of the ion features to a referent library of chemical standard records including preferred adducts, retention time, molecular weight (m/z), and in-source fragments in addition to related MS spectra. Also, the in-house software developed by Metabolon was used to implement the visual inspection for quality control (QC) in order to curate the metabolites [3,4]. The identification of recognized chemical entities was based on a comparison to purified standards' metabolomic library entries. The detectable properties of commercially available purified standard substances have been determined. For structurally unidentified biochemicals that have been recognized due to their recurrence, new mass spectral entries

have been established (both chromatographic and mass spectral). The area-under-the-curve method was used to calculate the peaks.

In total, 1,136 metabolites were discovered, including 782 known and 354 unknown metabolites. Finally, 640 analyzable metabolites were verified as only known metabolites with missing rates ≤ 25% were regarded for quality control. Missing data for the metabolites were imputed to half of the lowest value [5,6].

(a)

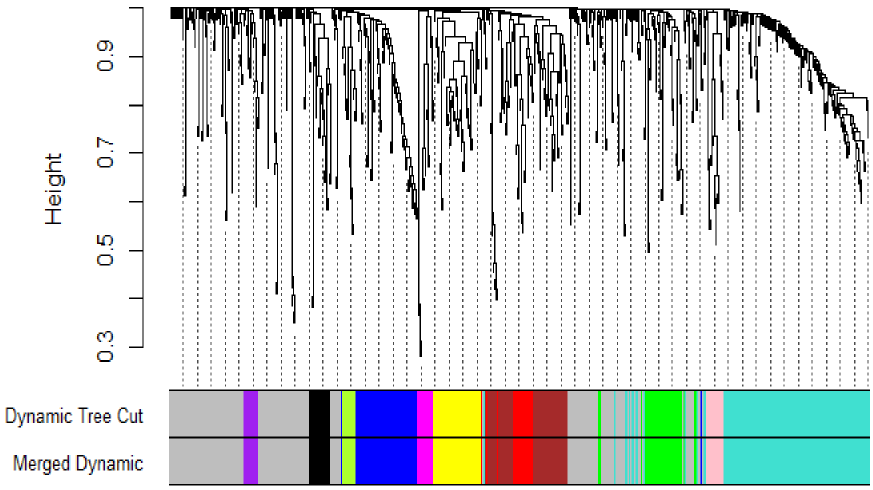

(b)

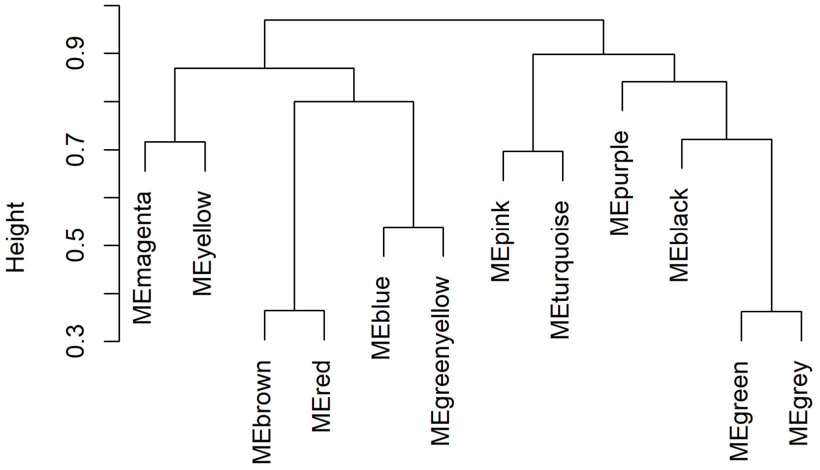

Figure S1. (a) Clustering dendrograms of metabolites, with dissimilarity based on topological overlap, along with assigned module colors. 12 co-expression colored modules were established; (b) eigengene dendrogram constructed by proximities of module

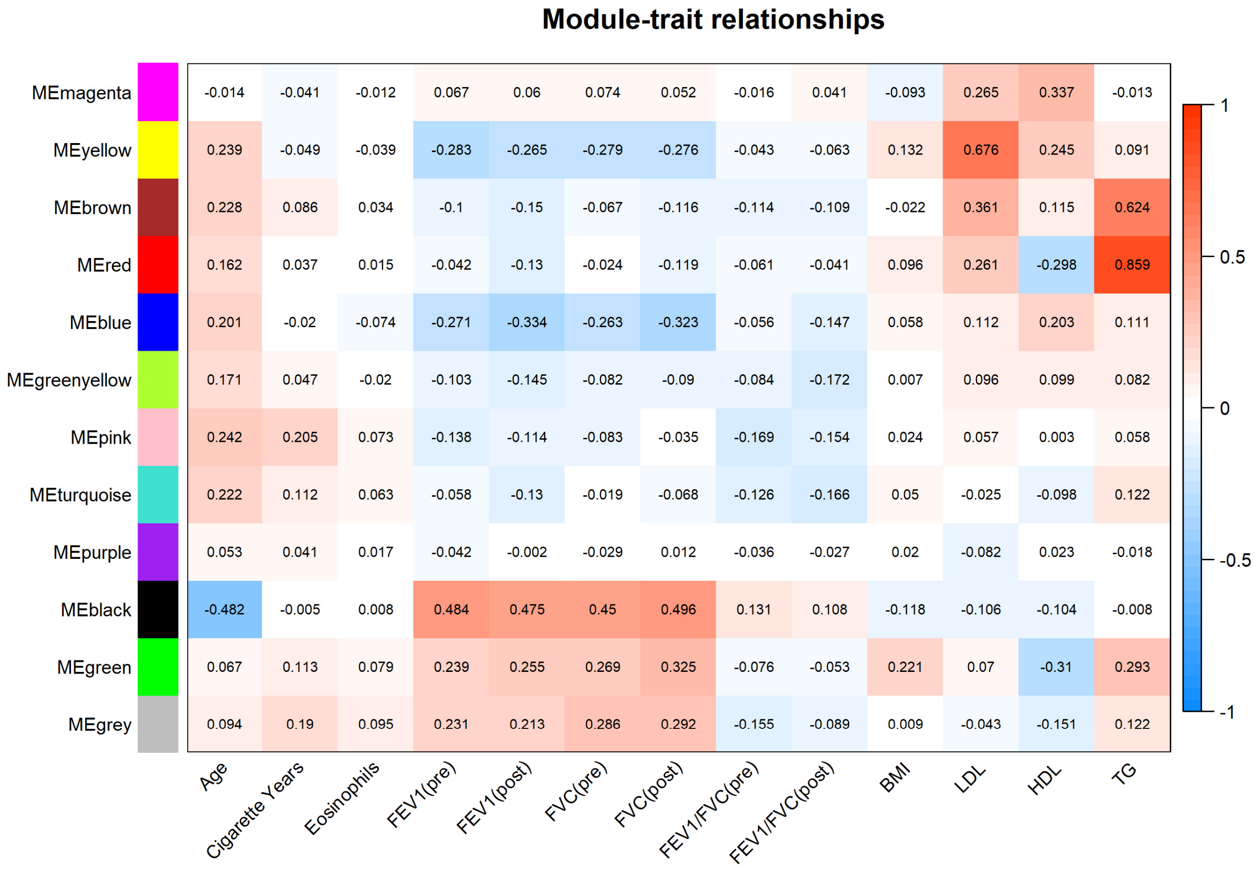

Figure S2. Module-trait relationship heatmap. Each row represents a module eigengene and each column to a trait. A stronger positive correlation between a module eigengene and a trait is displayed in darker red, and a stronger negative in darker blue.

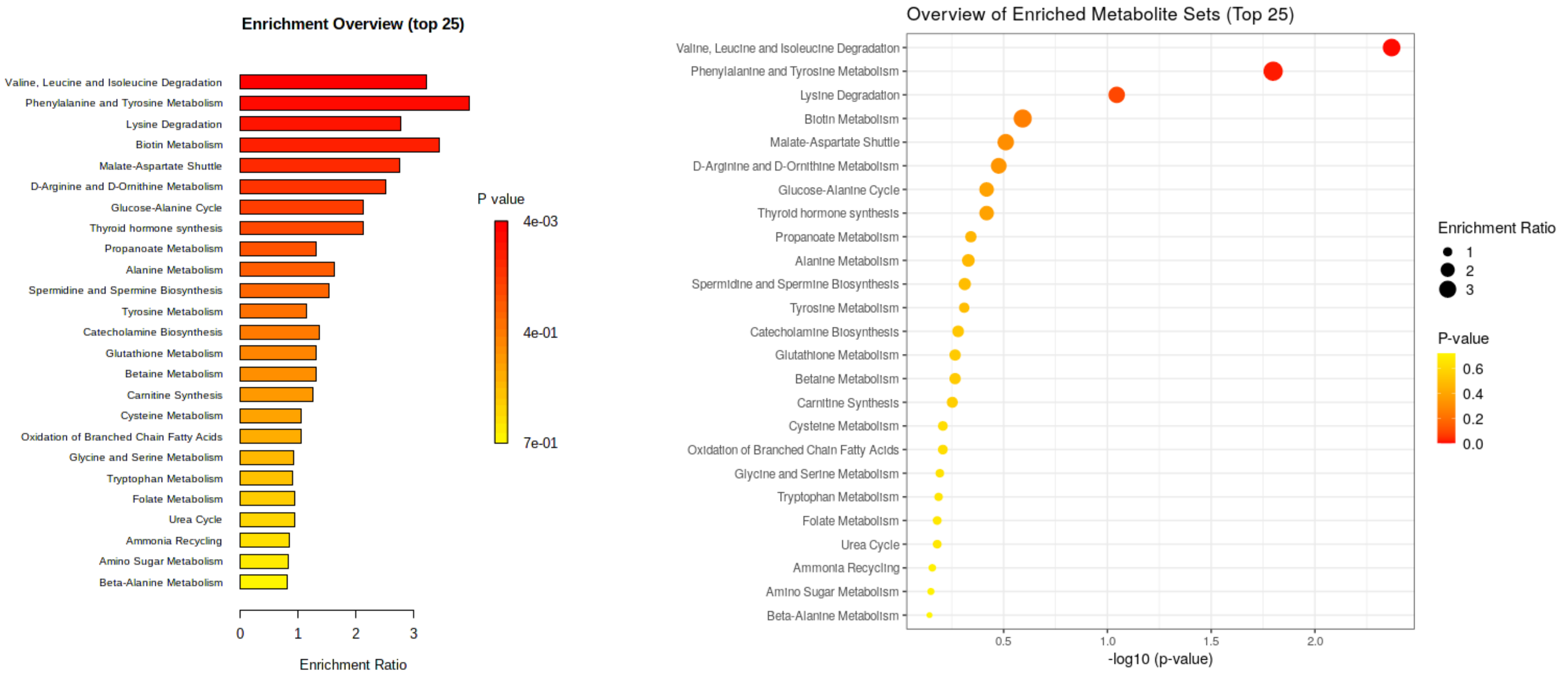

Figure S3. Over-representation analysis of the 40 metabolites in Green module

(a)

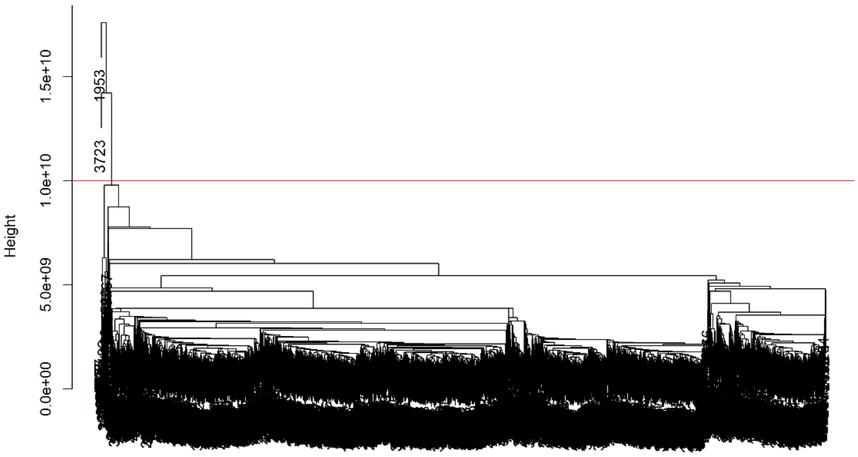

(b)

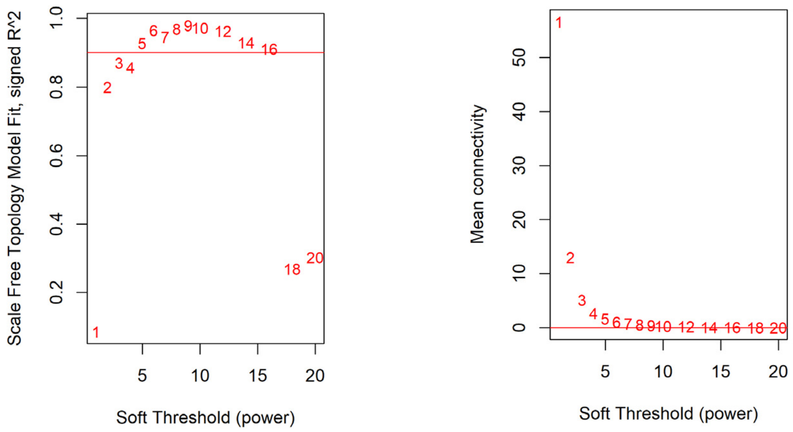

Figure S4. (a) Outliers detection by *SampleTree* function of WGCNA. Two outliers (1953, and 3723) were identified and removed; (b) analysis of network topology for a set of soft-thresholding powers

Table S1. Demographic Characteristics of the Samples in Study Used for the Heatmap of the Pearson Correlation Test (N = 3,347)

	**Asthma (N=514)**	**Non-asthma (N = 2,833)**	***p*-value**
**Cigarette Pack Years, Cigarette-Years**[1)]	9.34 ± 18.66	5.75 ± 13.90	<0.001
**Eosinophils, Counts**	0.22 ± 0.16	0.19 ± 0.18	<0.001
**Pulmonary Function Measures**			
pre FEV1, mL	2550 ± 836	2929 ± 785	<0.001
pre FVC, mL	3277 ± 376	3635 ± 980	<0.001
pre FEV1/FVC, %	77.4 ± 9.56	80.77 ± 6.36	<0.001
post FEV1, mL	2278 ± 902	2730 ± 800	<0.001
post FVC, mL	3393 ± 1136	3829 ± 990	0.004
post FEV1/FVC, %	66.7 ± 10.41	71.0 ± 8.27	0.001

[1)] The number of exposure years multiplied by the average number of cigarettes smoked per day

Table S2. The Number of Metabolites in 12 Modules

Module Colors	Frequency
Black	20
Blue	59
Brown	57
Green	40
Green-Yellow	13
Grey	191
Magenta	15
Pink	15
Purple	14
Red	21
Turquoise	151
Yellow	44
Total	640

Table S3. Association between Colored Modules and Asthma

Module	Model (1)	Model (2)	Model (3)	Model (4)
Green	1.28*	1.25	1.25	1.15
	(1.10, 1.49)	(1.07, 1.47)	(1.07, 1.46)	(0.97, 1.36)
Grey	1.14	1.15	1.10	1.12
	(0.96, 1.36)	(0.96, 1.37)	(0.93, 1.30)	(0.94, 1.33)
Green-yellow	1.07	1.08	1.09	1.09
	(0.95, 1.21)	(0.95, 1.22)	(0.96, 1.23)	(0.96, 1.23)
Red	1.10	1.01	1.02	1.02
	(0.96, 1.25)	(0.79, 1.28)	(0.80, 1.32)	(0.80, 1.31)
Purple	1.02	1.03	1.01	1.01
	(0.91, 1.14)	(0.92, 1.16)	(0.90, 1.13)	(0.90, 1.14)
Brown	1.04	1.00	0.98	1.01
	(0.91, 1.18)	(0.81, 1.23)	(0.80, 1.21)	(0.82, 1.24)
Pink	1.00	1.00	0.97	0.97
	(0.88, 1.14)	(0.88, 1.14)	(0.85, 1.10)	(0.86, 1.10)
Yellow	1.00	1.02	1.05	0.95
	(0.85, 1.17)	(0.84, 1.23)	(0.86, 1.27)	(0.78, 1.15)
Turquoise	0.95	0.93	0.91	0.93
	(0.84, 1.07)	(0.82, 1.05)	(0.80, 1.04)	(0.81, 1.05)
Blue	0.94	0.94	0.95	0.93
	(0.82, 1.07)	(0.82, 1.07)	(0.83, 1.08)	(0.81, 1.07)
Magenta	0.92	0.94	0.94	0.93
	(0.78, 1.09)	(0.79, 1.13)	(0.78, 1.13)	(0.79, 1.10)
Black	0.89	0.89	0.86	0.87
	(0.74, 1.07)	(0.74, 1.07)	(0.72, 1.03)	(0.73, 1.05)

Odds ratio with 95% confidence interval in parentheses

Bonferroni adjusted p-values: *** $p < 0.001$, ** $p < 0.01$, * $p < 0.05$

Model 1 included age, sex, immigration status, field center, years of living in the U.S., and Hispanic/Latino backgrounds; **Model 2** additionally adjusted for LDL, HDL, and TG; **Model 3** supplemented smoking, education level, and household income; and **Model 4** added BMI.

Table S4. Pathways and Metabolites Classification of Green Module

Super Pathways	Sub Pathways	Metabolites	Counts[1)]
Amino Acid	Glutamate Metabolism	glutamate*	1
	Glutathione Metabolism	2-aminobutyrate, 2-hydroxybutyrate/2-hydroxyisobutyrate	2
	Leucine, Isoleucine and Valine Metabolism	2-hydroxy-3-methylvalerate, 3-hydroxyisobutyrate, 3-methyl-2-oxobutyrate*, 3-methyl-2-oxovalerate*, 4-methyl-2-oxopentanoate, alpha-hydroxyisocaproate, alpha-hydroxyisovalerate, beta-hydroxyisovalerate, isoleucine*, isovalerylcarnitine (C5)*, leucine*, valine*	12
	Lysine Metabolism	2-aminoadipate*, lysine	2
	Methionine, Cysteine, SAM and Taurine Metabolism	methionine	1
	Phenylalanine Metabolism	phenylalanine*, phenyllactate (PLA)	2
	Tryptophan Metabolism	indolelactate, tryptophan*	2
	Tyrosine Metabolism	3-(4-hydroxyphenyl)lactate, 4-hydroxyphenylpyruvate*, tyrosine*	3
	Urea cycle; Arginine and Proline Metabolism	ornithine	1
Lipid	Fatty Acid Metabolism (also BCAA Metabolism)	propionylcarnitine (C3)	1
Nucleotide	Purine Metabolism, (Hypo)Xanthine/Inosine containing	urate	1
Peptide	Gamma-glutamyl Amino Acid	gamma-glutamyl-2-aminobutyrate, gamma-glutamyl-alpha-lysine, gamma-glutamyl-epsilon-lysine, gamma-glutamylglutamate*, gamma-glutamylhistidine, gamma-glutamylisoleucine*, gamma-glutamylleucine*, gamma-glutamylmethionine, gamma-glutamylphenylalanine*, gamma-glutamyltryptophan*, gamma-glutamyltyrosine*, gamma-glutamylvaline*	12

1) In total, 40 metabolites are in Green module.

* Metabolites whose raw *p*-values are less than 0.05 on Model 3 of the single metabolite analysis

Table S5. Over-Representation Analysis of the 40 Metabolites in Green Module

	total	expected	hits	Raw p	Holm p	FDR
Valine, Leucine and Isoleucine Degradation	60	2.17	7	0.00427	0.418	0.418
Phenylalanine and Tyrosine Metabolism	28	1.01	4	0.0159	1	0.777
Lysine Degradation	30	1.08	3	0.0902	1	1
Biotin Metabolism	8	0.289	1	0.256	1	1
Malate-Aspartate Shuttle	10	0.361	1	0.309	1	1
D-Arginine and D-Ornithine Metabolism	11	0.397	1	0.334	1	1
Glucose-Alanine Cycle	13	0.47	1	0.382	1	1
Thyroid hormone synthesis	13	0.47	1	0.382	1	1
Propanoate Metabolism	42	1.52	2	0.455	1	1
Alanine Metabolism	17	0.614	1	0.468	1	1
Spermidine and Spermine Biosynthesis	18	0.65	1	0.487	1	1
Tyrosine Metabolism	72	2.6	3	0.49	1	1
Catecholamine Biosynthesis	20	0.723	1	0.524	1	1
Glutathione Metabolism	21	0.759	1	0.542	1	1
Betaine Metabolism	21	0.759	1	0.542	1	1
Carnitine Synthesis	22	0.795	1	0.559	1	1
Cysteine Metabolism	26	0.939	1	0.621	1	1
Oxidation of Branched Chain Fatty Acids	26	0.939	1	0.621	1	1
Glycine and Serine Metabolism	59	2.13	2	0.642	1	1
Tryptophan Metabolism	60	2.17	2	0.651	1	1
Folate Metabolism	29	1.05	1	0.661	1	1
Urea Cycle	29	1.05	1	0.661	1	1
Ammonia Recycling	32	1.16	1	0.698	1	1
Amino Sugar Metabolism	33	1.19	1	0.709	1	1
Beta-Alanine Metabolism	34	1.23	1	0.72	1	1
Aspartate Metabolism	35	1.26	1	0.73	1	1
Nicotinate and Nicotinamide Metabolism	37	1.34	1	0.75	1	1
Purine Metabolism	74	2.67	2	0.764	1	1
Methionine Metabolism	43	1.55	1	0.801	1	1
Histidine Metabolism	43	1.55	1	0.801	1	1
Glutamate Metabolism	49	1.77	1	0.842	1	1
Arginine and Proline Metabolism	53	1.92	1	0.865	1	1
Warburg Effect	58	2.1	1	0.889	1	1
Arachidonic Acid Metabolism	69	2.49	1	0.928	1	1

Table S6. Stratification Analysis of Green module and 1-arachidonoyl-GPA (20:4) by Sex and Hispanic/Latino Backgrounds

		Cases/Controls	Model (1)	Model (2)	Model (3)	Model (4)
Sex						
Green Module	Female	343/1561	1.41*** (1.22, 1.64)	1.36*** (1.17, 1.59)	1.37*** (1.17, 1.60)	1.27** (1.08, 1.49)
	Male	171/1272	1.00 (0.83, 1.21)	1.05 (0.87, 1.27)	1.04 (0.86, 1.25)	1.04 (0.85, 1.26)
1-arachidonoyl-GPA (20:4)	Female	343/1561	1.23*** (1.09, 1.38)	1.22*** (1.08, 1.37)	1.24*** (1.10, 1.40)	1.21** (1.07, 1.37)
	Male	171/1272	1.12 (0.95, 1.32)	1.15 (0.96, 1.35)	1.13 (0.95, 1.34)	1.13 (0.95, 1.34)
Hispanic/Latino Backgrounds						
Green Module	Cuban and Puerto-Rican Backgrounds	317/849	1.27** (1.09, 1.47)	1.26** (1.08, 1.48)	1.27** (1.09, 1.49)	1.24** (1.06, 1.47)
	Others	197/1984	1.21* (1.02, 1.45)	1.20* (1.00, 1.44)	1.20 (1.00, 1.44)	1.10 (0.90, 1.33)
1-arachidonoyl-GPA (20:4)	Cuban and Puerto-Rican Backgrounds	317/849	1.26*** (1.10, 1.44)	1.26*** (1.10, 1.44)	1.25** (1.09, 1.43)	1.24** (1.08, 1.42)
	Others	197/1984	1.14 (0.99, 1.30)	1.15* (1.00, 1.31)	1.15* (1.00, 1.32)	1.13 (0.98, 1.29)

Odds ratio with 95% confidence interval in parentheses

*** $p < 0.001$, ** $p < 0.01$, * $p < 0.05$

Model 1 included age, sex, immigration status, field center, years of living in the U.S., and Hispanic/Latino backgrounds; **Model 2** additionally adjusted for LDL, HDL, and TG; **Model 3** supplemented smoking, education level, and household income; and **Model 4** added BMI.

Table S7. Interaction Effects of Green module and 1-arachidonoyl-GPA (20:4) by Sex and Hispanic/Latino Backgrounds

	Sex						Hispanic/Latino Backgrounds					
	Men		Women		z score	p-value	Cuban and Puerto-Rican Backgrounds		Others		z score	p-value
Model	beta	s.e.	beta	s.e.			beta	s.e.	beta	s.e.		
**Green Module**												
1	0.004	0.094	0.346	0.075	113.53	<0.001	0.236	0.094	0.194	0.075	13.23	<0.001
2	0.049	0.097	0.310	0.079	83.74	<0.001	0.233	0.080	0.184	0.093	16.05	<0.001
3	0.035	0.097	0.313	0.079	88.52	<0.001	0.243	0.080	0.184	0.094	18.96	<0.001
4	0.036	0.100	0.239	0.082	62.65	<0.001	0.219	0.083	0.094	0.100	38.73	<0.001
**1-arachidonoyl-GPA (20:4)**												
1	0.116	0.085	0.204	0.060	33.56	<0.001	0.227	0.068	0.130	0.070	38.76	<0.001
2	0.136	0.087	0.199	0.060	23.51	<0.001	0.228	0.069	0.138	0.070	35.96	<0.001
3	0.121	0.088	0.214	0.062	34.46	<0.001	0.223	0.070	0.142	0.071	31.75	<0.001
4	0.121	0.088	0.194	0.062	27.10	<0.001	0.214	0.070	0.120	0.072	36.40	<0.001

Model 1 included age, sex, immigration status, field center, years of living in the U.S., and Hispanic/Latino backgrounds; **Model 2** additionally adjusted for LDL, HDL, and TG; **Model 3** supplemented smoking, education level, and household income; and **Model 4** added BMI.

Table S8. The List of 1-arachidonoyl-GPA (20:4) and 40 Metabolites in Green Module by LC/MS Analysis

Metabolite	Platform	RI	MASS	CAS ID	PUB CHEM	KEGG	HMDB	SMILES
1-arachidonoyl-GPA (20:4)	LC/MS Neg	5499	457.2361	799268-65-8	NA	NA	NA	CCCCC\C=C/C/C=C\C/C=C\C/C=C\CCCC(OCC(O)COP(O)(O)=O)=O
2-aminoadipate	LC/MS Neg	639.2	160.0615	542-32-5; 1118-90-7	469	C00956	HMDB00510	OC(CCCC(N)C(O)=O)=O
2-aminobutyrate	LC/MS Pos Early	2059	104.0706	1492-24-6	439691	C02261	HMDB00650	N[C@H](CC)C(O)=O
2-hydroxy-3-methylvalerate	LC/MS Neg	1800	131.0714	488-15-3	164623	NA	HMDB00317	O=C(C(C(CC)C)O)O
2-hydroxybutyrate/2-hydroxyisobutyrate	LC/MS Polar	1258	103.0401	NA	NA	NA	NA	CCC(O)C(O)=O
3-(4-hydroxyphenyl)lactate	LC/MS Neg	1379	181.0506	6482-98-0	9378	C03672	HMDB00755	OC(C(O)=O)CC1=CC=C(O)C=C1
3-hydroxyisobutyrate	LC/MS Polar	1619	103.0401	2068-83-9	87	C06001	HMDB00336	CC(C(O)=O)CO
3-methyl-2-oxobutyrate	LC/MS Neg	1465	115.0401	3715-29-5	49	C00141	HMDB00019	OC(C(C(C)C)=O)=O
3-methyl-2-oxovalerate	LC/MS Neg	2064.2	129.0557	1460-34-0; 51829-07-3	47	C00671	HMDB03736	CCC(C)C(C(O)=O)=O
4-hydroxyphenylpyruvate	LC/MS Neg	1690	179.035	156-39-8	979	C01179	HMDB00707	OC1=CC=C(CC(C(O)=O)=O)C=C1
4-methyl-2-oxopentanoate	LC/MS Neg	2170	129.0557	816-66-0	70	C00233	HMDB00695	CC(C)CC(C(O)=O)=O
alpha-hydroxyisocaproate	LC/MS Neg	1840	131.0714	10303-64-7	83697	C03264	HMDB00746	OC(C(CC(C)C)O)=O
alpha-hydroxyisovalerate	LC/MS Polar	1052	117.0557	600-37-3	99823	NA	HMDB00407	OC(C(O)=O)C(C)C
beta-hydroxyisovalerate	LC/MS Neg	1027	117.0557	625-08-1	69362	NA	HMDB00754	CC(C)(O)CC(O)=O
gamma-glutamyl-2-aminobutyrate	LC/MS Pos Early	2380	233.1132	16869-42-4	NA	NA	NA	CCC(C(=O)[O-])NC(=O)CCC(C(=O)[O-])[NH3+]
gamma-glutamyl-alpha-lysine	LC/MS Pos Early	2784	276.1554	NA	65254	NA	NA	O=C(N[C@@H](CCCCN)C(O)=O)CC[C@H](N)C(O)=O
gamma-glutamyl-epsilon-lysine	LC/MS Pos Early	2717	276.1554	17105-15-6	7015685	NA	HMDB03869	N[C@H](C(O)=O)CCCCNC(CC[C@@H](C(O)=O)N)=O
gamma-glutamylglutamate	LC/MS Pos Early	1775	277.103	1116-22-9	92865	C05282	HMDB11737	O=C(O)C(N)CCC(NC(C(O)=O)CCC(O)=O)=O
gamma-glutamylhistidine	LC/MS Pos Early	2740	285.1194	37460-15-4	7017195	NA	NA	O=C(O)[C@@H](N)CCC(N[C@@H](CC1=CNC=N1)C(O)=O)=O
gamma-glutamylisoleucine*	LC/MS Pos Early	2940	261.1445	NA	14253342	NA	HMDB11170	CCC(C)[C@@H](C(O)=O)NC(CC[C@@H](C(O)=O)N)=O

gamma-glutamylleucine	LC/MS Pos Early	2991	261.1445	2566-39-4	151023	NA	HMDB11171	CC(C)C[C@@H](C(O)=O)NC(CC[C@@H](C(O)=O)N)=O
gamma-glutamylmethionine	LC/MS Pos Early	2640	279.1009	17663-87-5	7009567	NA	HMDB29155	O=C(O)[C@H](CCSC)NC(CC[C@@H](C(O)=O)N)=O
gamma-glutamylphenylalanine	LC/MS Pos Early	2992	295.1289	7432-24-8	111299	NA	HMDB00594	O=C(O)[C@H](CC1=CC=CC=C1)NC(CC[C@@H](C(O)=O)N)=O
gamma-glutamyltryptophan	LC/MS Pos Early	2975	334.1398	66471-20-3	3989307	NA	HMDB29160	O=C(O)[C@@H](N)CCC(N[C@@H](CC1=CNC2=C1C=CC=C2)C(O)=O)=O
gamma-glutamyltyrosine	LC/MS Neg	1240	309.1092	7432-23-7	94340	NA	HMDB11741	OC(C=C1)=CC=C1C[C@@H](C(O)=O)NC(CC[C@@H](C(O)=O)N)=O
gamma-glutamylvaline	LC/MS Pos Early	2700	247.1289	2746-34-1	7015683	NA	HMDB11172	O=C(N[C@H]([C@@](O)=O)C(C)C)CC[C@@H]([C@](O)=O)N
glutamate	LC/MS Pos Early	1500	148.0604	56-86-0	611	C00025	HMDB00148	O=C(O)[C@@H](N)CCC(O)=O
indolelactate	LC/MS Neg	2286	204.0666	832-97-3	92904	C02043	HMDB00671	OC(C(O)=O)CC1=CNC2=C1C=CC=C2
isoleucine	LC/MS Pos Early	2800	132.1019	73-32-5	6306	C00407	HMDB00172	NC(C(O)=O)C(C)CC
isovalerylcarnitine (C5)	LC/MS Pos Early	3085	246.17	31023-24-2	6426851	NA	HMDB00688	[O-]C(CC(C[N+](C)(C)C)OC(CC(C)C)=O)=O
leucine	LC/MS Pos Early	2864	132.1019	61-90-5	6106	C00123	HMDB00687	O=C([C@H](CC(C)C)N)O
lysine	LC/MS Pos Early	2850	147.1128	56-87-1	5962	C00047	HMDB00182	NC(C(O)=O)CCCCN
methionine	LC/MS Pos Early	2526	150.0583	63-68-3	6137	C00073	HMDB00696	CSCCC(N)C(O)=O
ornithine	LC/MS Pos Early	2800	133.0972	3184-13-2	6262	C00077	HMDB03374	O=C(O)[C@@H](N)CCCN
phenylalanine	LC/MS Pos Early	2878	166.0863	63-91-2	6140	C00079	HMDB00159	NC(C(O)=O)CC1=CC=CC=C1
phenyllactate (PLA)	LC/MS Neg	2208	165.0557	828-01-3	3848	C05607	HMDB00779	OC(C(O)=O)CC1=CC=CC=C1
propionylcarnitine (C3)	LC/MS Pos Early	2590	218.1387	17298-37-2	107738	C03017	HMDB00824	[O-]C(CC(OC(CC)=O)C[N+](C)(C)C)=O
tryptophan	LC/MS Pos Early	2986	205.0972	73-22-3	6305	C00078	HMDB00929	O=C(O)[C@@H](N)CC1=CNC2=C1C=CC=C2
tyrosine	LC/MS Pos Early	2430	182.0812	60-18-4	6057	C00082	HMDB00158	N[C@@H](CC1=CC=C(O)C=C1)C(O)=O
urate	LC/MS Neg	757.1	167.0211	69-93-2; 120K5305	1175	C00366	HMDB00289	O=C1NC(NC(NC2=O)=O)=C2N1
valine	LC/MS Pos Early	2479	118.0863	72-18-4	6287	C00183	HMDB00883	N[C@H](C(O)=O)C(C)C

RI: Relative intensity; CAS ID: Chemical Abstracts Service Identification number; SMILE: SMall Incision Lenticule Extraction

Table S9. Scale Free Metrics Resulting from *pickSoftThreshold* Function of WGCNA

	Power	SFT.R.sq	slope	truncated.R.sq	mean.k.	median.k.	max.k.
1	1	0.086	-0.478	0.872	56.6	55.1	117
2	2	0.8	-1.18	0.975	13	10.6	48.1
3	3	0.87	-1.38	0.89	5.15	3.39	28.7
4	4	0.858	-1.47	0.837	2.71	1.48	19.4
5	5	0.928	-1.39	0.917	1.66	0.726	13.8
6	6	0.965	-1.36	0.956	1.11	0.428	10.2
7	7	0.945	-1.38	0.933	0.781	0.254	7.91
8	8	0.969	-1.36	0.961	0.575	0.165	6.21
9	9	0.979	-1.33	0.974	0.437	0.106	4.93
10	10	0.972	-1.34	0.967	0.34	0.0684	4.08
11	12	0.963	-1.36	0.965	0.218	0.0337	3.04
12	14	0.929	-1.43	0.94	0.148	0.0171	2.38
13	16	0.911	-1.43	0.916	0.105	0.00769	1.96
14	18	0.269	-2.17	0.117	0.0773	0.00361	1.67
15	20	0.303	-2.2	0.239	0.0586	0.00173	1.46

Supplementary reference

1. Feofanova, E.V.; Chen, H.; Dai, Y.; Jia, P.; Grove, M.L.; Morrison, A.C.; Qi, Q.; Daviglus, M.; Cai, J.; North, K.E.; et al. A Genome-wide Association Study Discovers 46 Loci of the Human Metabolome in the Hispanic Community Health Study/Study of Latinos. *Am J Hum Genet* **2020**, *107*, 849-863, doi:10.1016/j.ajhg.2020.09.003.
2. Evans AM, B.B., , Liu Q, , Mitchell MW, , Robinson RJ, , Dai H, Stewart SJ, , DeHaven CD, and Miller LAD. High Resolution Mass Spectrometry Improves Data Quantity and Quality as Compared to Unit Mass Resolution Mass Spectrometry in HighThroughput Profiling Metabolomics. *Journal of Postgenomics Drug & Biomarker Development* **2014**, *4*, doi:10.4172/2153-0769.1000132.
3. Chen, G.C.; Chai, J.C.; Yu, B.; Michelotti, G.A.; Grove, M.L.; Fretts, A.M.; Daviglus, M.L.; Garcia-Bedoya, O.L.; Thyagarajan, B.; Schneiderman, N.; et al. Serum sphingolipids and incident diabetes in a US population with high diabetes burden: the Hispanic Community Health Study/Study of Latinos (HCHS/SOL). *Am J Clin Nutr* **2020**, *112*, 57-65, doi:10.1093/ajcn/nqaa114.
4. Dehaven, C.D.; Evans, A.M.; Dai, H.; Lawton, K.A. Organization of GC/MS and LC/MS metabolomics data into chemical libraries. *J Cheminform* **2010**, *2*, 9, doi:10.1186/1758-2946-2-9.
5. Wei, R.; Wang, J.; Su, M.; Jia, E.; Chen, S.; Chen, T.; Ni, Y. Missing Value Imputation Approach for Mass Spectrometry-based Metabolomics Data. *Sci Rep* **2018**, *8*, 663, doi:10.1038/s41598-017-19120-0.
6. Playdon, M.C.; Joshi, A.D.; Tabung, F.K.; Cheng, S.; Henglin, M.; Kim, A.; Lin, T.; van Roekel, E.H.; Huang, J.; Krumsiek, J.; et al. Metabolomics Analytics Workflow for Epidemiological Research: Perspectives from the Consortium of Metabolomics Studies (COMETS). *Metabolites* **2019**, *9*, doi:10.3390/metabo9070145.
